# AFTM: a database of transmembrane regions in the human proteome predicted by AlphaFold

**DOI:** 10.1093/database/baad008

**Published:** 2023-03-14

**Authors:** Jimin Pei, Qian Cong

**Affiliations:** Eugene McDermott Center for Human Growth and Development, University of Texas Southwestern Medical Center, 6001 Forest Park Rd, Dallas, TX 75390, USA; Department of Biophysics, University of Texas Southwestern Medical Center, 6001 Forest Park Rd, Dallas, TX 75390, USA; Harold C. Simmons Comprehensive Cancer Center, University of Texas Southwestern Medical Center, 6001 Forest Park Rd., Dallas, TX 75390, USA; Eugene McDermott Center for Human Growth and Development, University of Texas Southwestern Medical Center, 6001 Forest Park Rd, Dallas, TX 75390, USA; Department of Biophysics, University of Texas Southwestern Medical Center, 6001 Forest Park Rd, Dallas, TX 75390, USA; Harold C. Simmons Comprehensive Cancer Center, University of Texas Southwestern Medical Center, 6001 Forest Park Rd., Dallas, TX 75390, USA

## Abstract

Transmembrane proteins (TMPs), with diverse cellular functions, are difficult targets for structural determination. Predictions of TMPs and the locations of transmembrane segments using computational methods could be unreliable due to the potential for false positives and false negatives and show inconsistencies across different programs. Recent advances in protein structure prediction methods have made it possible to identify TMPs and their membrane-spanning regions using high-quality structural models. We developed the AlphaFold Transmembrane proteins (AFTM) database of candidate human TMPs by identifying transmembrane regions in AlphaFold structural models of human proteins and their domains using the positioning of proteins in membranes, version 3 program, followed by automatic corrections inspired by manual analysis of the results. We compared our results to annotations from the UniProt database and the Human Transmembrane Proteome (HTP) database. While AFTM did not identify transmembrane regions in some single-pass TMPs, it identified more transmembrane regions for multipass TMPs than UniProt and HTP. AFTM also showed more consistent results with experimental structures, as benchmarked against the Protein Data Bank Transmembrane proteins (PDBTM) database. In addition, some proteins previously annotated as TMPs were suggested to be non-TMPs by AFTM. We report the results of AFTM together with those of UniProt, HTP, TmAlphaFold, PDBTM and Membranome in the online AFTM database compiled as a comprehensive resource of candidate human TMPs with structural models.

**Database URL** http://conglab.swmed.edu/AFTM

## Introduction

Transmembrane proteins (TMPs) constitute a significant portion of the human proteome, accounting for ∼20–30% of all proteins (1). These proteins play vital roles in a variety of cellular functions, such as transport, signal transduction, energy production and immune response (2, 3). As TMPs are the main class of proteins targeted by drugs, understanding their structures and how they function is crucial for drug discovery (4, 5). Recent advances in structural determination techniques have led to a significant increase in the number of TMPs with known structures (6, 7). To catalog these membrane-associated proteins, several structural databases have been developed, including Protein Data Bank Transmembrane proteins (PDBTM) (8), Orientations of Proteins in Membranes (9), mpstruc (10) and MemProtMD (11).

Accurate identification of transmembrane segments (TMSs) is essential for determining the membrane topology of TMPs, which has practical applications in drug design. Several programs have been developed to recognize TMSs in experimentally determined 3D structures of proteins (8, 12, 13). For TMPs without experimental structures, there are also many programs that can predict the location of TMSs in their primary sequences. However, these computational predictions of TMSs, often based on hydrophobic propensities and evolutionary information, can sometimes be inaccurate with false positives (incorrectly identifying a segment as a TMS) and false negatives (failing to identify a TMS). This can occur, for example, when a hydrophobic α-helical segment embedded in the core of a soluble domain is incorrectly predicted as a TMS or when a TMS with several hydrophilic residues is missed by computational programs. It is common to see discrepancies between the results of different TMS prediction methods when they are compared (1, 13).

The UniProt database (14) provides annotations of TMPs and their TMSs in the human proteome. The Human Transmembrane Proteome (HTP) database (13) offers another comprehensive resource for human TMPs and their TMSs, combining information from various sources such as available structures, experimental studies of membrane topology, and consensus predictions from multiple TMS prediction programs. The recent advancements in protein structure prediction through AlphaFold (15), as evidenced in the Critical Assessment of Structure Prediction, 14th experiment (16), have greatly improved our understanding of the structures of human proteins (17). In this study, we predicted TMSs from AlphaFold models of human proteins and compared the results to the annotations of UniProt and HTP. We present our results in the AFTM online database that provides a comprehensive resource of potential human TMPs annotated by various sources.

## Results and Discussion

### AFTM—defining TMPs and their TMSs in AlphaFold models

We used the positioning of proteins in membranes, version 3 (PPM3) method (18) to predict the localizations of TMSs in AlphaFold structural models of the human proteome. This dataset includes 5491 potential human TMPs compiled from a combination of reviewed UniProt entries (23 February 2022) with annotated TMSs and entries annotated as TMPs in the HTP database (13). PPM3 was developed to identify TMSs in experimental structures, and we made several modifications to adapt it for AlphaFold models. These changes include removing signal peptides and mitochondrial transit peptides, removing disordered regions for prediction of TMSs from full-length proteins, partitioning proteins into domains for domain-based TMS predictions, differentiating transmembrane and re-entrant regions, joining discontinuous TMS regions and identifying some missing TMSs based on the orientation of reported TMSs (see details in the Materials and methods section and Supplementary Figure S1).

### Comparison of TMPs defined by AFTM, UniProt and HTP

**Figure 1.**
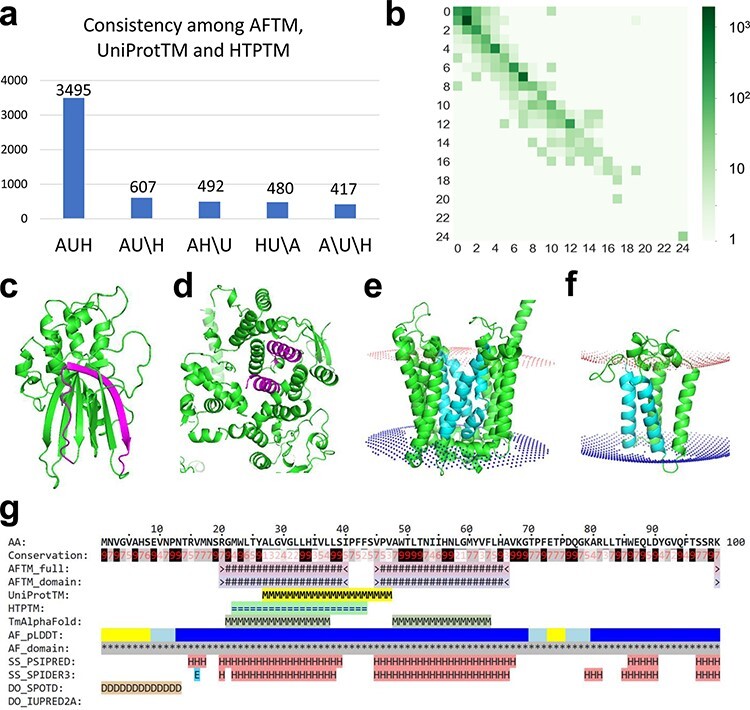
(a) Statistics of comparisons of TMSs annotated by AFTM (A), UniProt (U) and HTP (H). AUH: TMSs are consistent among the three methods. AU\H: TMSs are consistent between AFTM and UniProt, but inconsistent compared to HTP. The same annotation is applied for AH\U and HU\A. A\U\H: TMSs are inconsistent among all three methods. (b) Comparison between the number of TMSs reported by AFTM and UniProt. Each square represents the number of proteins with a certain number of TMSs predicted by AFTM (*X*-axis) and a certain number of TMSs reported by UniProt (*Y*-axis). The numbers are colored according to a 10-based logarithm of the counts of proteins. (c, d) AlphaFold models of TMEM183A (accession number: Q8IXX5) (c) and UBR3 (accession number: Q6ZT12 (d), with potential false TMSs by UniProt and HTP colored in magenta. (e, f) AlphaFold models of ANKH (accession number: Q9HCJ1) (e) and ORMDL1 (accession number: Q9P0S3) (f), with missing TMSs by UniProt and HTP colored in cyan. (g) A sequence block of ORMDL1 from the AFTM website.

We compared the TMSs defined by AFTM to those reported in UniProt and HTP. We considered two TMSs as consistently annotated (matched) if they overlap for ≥10 residues, and we considered a protein to be consistently annotated by different resources if all their TMSs are matched. Of the 5491 TMPs, 3495 (64%) have consistent annotations regarding the locations of TMSs by AFTM, UniProt and HTP. An additional 1579 (29%) proteins are consistently annotated between two resources (Figure 1a).

The AFTM procedure missed ∼20% of single-pass TMPs compared to UniProt or HTP (Figure 1b; Supplementary Tables S1 and S2). Bcl-2 homologous antagonist/killer (BAK1), Junctophilin-3 (JPH3), Potassium voltage-gated channel subfamily E member 1 (KCNE1), Killer cell lectin-like receptor subfamily K member (1KLRK1), Kelch domain-containing protein 7A (KLHDC7A) and Phosphoglycerate mutase family member 5 (PGAM5)are examples of single-pass TMPs annotated by UniProt, HTP and the curated database Membranome (19), while AFTM reported no TMSs. Manual inspections of the structural models revealed that single hydrophobic transmembrane helix may not be reliably predicted by AlphaFold (showing low predicted Local Distance Difference Test (pLDDT) scores). In addition, because AlphaFold models of single-pass TMPs could have extracellular domains, intracellular domains and transmembrane regions intertwined due to the flexibility of the linker regions, PPM3 often failed to identify the TMSs for these proteins. These problems were also recognized in the application of AlphaFold models in finding TMSs in Membranome, the curated database of single-pass TMPs in several organisms including humans (19). Thus, Membranome has relied on its in-house program (D-linker) to separate extracellular, transmembrane and intracellular regions in AlphaFold models in order to optimize their spatial placements relative to the membrane and improve the identification of TMSs in single-pass TMPs (19).

We used the entries in the Membranome database as a benchmark to evaluate the accuracy of AFTM, UniProt and HTP in predicting the presence and location of TMSs in single-pass TMPs (Table 1). Of the 2322 human single-pass TMPs defined in Membranome and mapped to our dataset, we observed that UniProt gave the most consistent results compared to the benchmark (Table 1), with 2196 proteins predicted to have a single TMS located in the same region as reported in Membranome. AFTM has the highest number of misses with 309 Membranome-defined single-pass TMPs not predicted to have a TMS, as compared to UniProt (69 proteins without TMS) and HTP (141 proteins without TMS). HTP has the highest number of cases (159 proteins) of predicting two or more TMSs for Membranome-defined single-pass TMPs, as compared to 61 cases for AFTM and 59 cases for UniProt. To aid in the identification of single-pass TMPs, we have included the results of Membranome in the AFTM online database, both in the main table and in the web pages of individual proteins.

**Table 1.** The performance of AFTM, UniProtTM and HTPTM on 2322 single-pass TMPs in the Membranome database

	no_TM	singleTM_match	singleTM_unmatch	multiple_TM
AFTM	309	1940	12	61
UniProtTM	69	2196	11	46
HTPTM	141	2009	13	159

no_TM: the number of proteins with no TMSs in AFTM, UniProtTM or HTPTM; singleTM_match: the number of proteins with a single TMS in AFTM, UniProtTM or HTPTM that matches the single TMS reported in Membranome; singleTM_unmatch: the number of proteins with a single TMS in AFTM, UniProtTM or HTPTM that does not match the region of the single TMS reported in Membranome; multiple_TM: the number of proteins with two or more TMSs in AFTM, UniProtTM or HTPTM.

Manual inspections revealed cases where AFTM did not predict the presence of TMSs in proteins that could be incorrectly annotated as TMPs by UniProt and HTP. For example, the protein E3 ubiquitin-protein ligase (UBR3) (20) was annotated to have three TMSs by UniProt (residues: 116–131, 764–779 and 919–934) and by HTP (residues: 761–781, 919–939 and 1806–1826), but AFTM suggests that it does not possess any TMSs. The analysis of the AlphaFold structural model of this protein revealed the presence of α-helical repeats with several α-helices buried in the core of the structure ( Figure 1d), which could explain the incorrect annotations by UniProt and HTP. Another example is a protein of unknown function named TMEM183A, which has a region (residues 300–320) predicted to be a TMS by UniProt and HTP, but not by AFTM. The analysis of the AlphaFold model revealed that part of this region corresponds to a hydrophobic β-strand (Figure 1c) within a soluble globular domain, suggesting that TMEM183A may not be a TMP despite its name. Such information could prove useful in future experimental studies of this protein.

AFTM tends to identify more TMSs for multipass TMPs compared to UniProt and HTP (Figure 1b; Supplementary Tables S1 and S2). Two such examples are shown in Figure 1e and f. The protein progressive ankylosis protein homolog (gene name: *ANKH*) (21) was predicted to have 12 tightly packed TMSs by AlphaFold, four of which (colored cyan) are missed by both UniProt and HTP. Another example is ORM1-like protein 1 (ORMDL1) (22), which was predicted to adopt a four-helical bundle fold with four TMSs identified by AFTM, but had only two TMSs annotated by UniProt and HTP (the potentially missed TMSs are shown in cyan in Figure 1f). Similar results were also observed for its two paralogs ORMDL2 and ORMDL3, where AFTM predicted four TMSs compared to one or two TMSs annotated by UniProt and HTP.

The TmAlphaFold database has been recently developed to visualize TMPs identified by AlphaFold models in multiple organisms, including humans (23). The main difference between TmAlphaFold and AFTM lies in the methods of placing protein structural models in the membrane. TmAlphaFold utilizes a simple geometric method named TMDET (24) that constructs planar membranes for potential TMPs. AFTM employs the recently developed PPM3 program (18) for protein-membrane placement. PPM3 takes into account the potential curvature of membranes and different membrane types and optimizes the free energy of transferring a protein from water to the membrane environment (18).

To evaluate the performance of AFTM, UniProtTM, Human Transmembrane Proteome transmembrane proteins (HTPTM) and TmAlphaFold, we compared their TMS predictions to those derived from experimental structures in the PDBTM database (25). A total of 601 human proteins in our dataset were mapped to at least one entry (represented by a PDB ID and the chain ID) in the PDBTM database with sequence identity >95% (see the Material and methods section). For each of these proteins, transmembrane regions in mapped PDBTM entries were combined to define TMSs of experimental structures (see the Materials and methods section). These TMSs were compared to those defined by AFTM, UniProtTM, HTPTM and TmAlphaFold. AFTM had the best performance in terms of missing PDBTM-defined TMSs, with only 66 TMSs defined in PDBTM not being predicted by AFTM, compared to 107 TMSs missed by TmAlphaFold, 197 TMSs missed by UniProtTM and 177 TMSs missed by HTPTM. One example of TMS missed by AFTM is the protein Acid-Sensing Ion Channel 1 (ASIC1), which has two TMSs reported in experimental structures (26, 27). Examination of the PPM3 output models revealed that both TMSs were embedded in the membrane. However, only the N-terminal TMS was reported by PPM3. This miss of the C-terminal TMS by PPM3 could be due to the broken and tilted transmembrane helix. AFTM also identified less than two TMSs in three other acid-sensing ion channel members (ASIC2, ASIC3 and ASIC5) and identified two TMSs in ASIC4.

AFTM identified ∼30 human proteins with predicted TMSs not reported by HTP or UniProt. Manual inspection of these proteins revealed that some of them could be true TMPs based on their homology to known TMPs or experimental evidence. For example, the N-terminal hydrophobic segments in several cytochrome P450 family proteins such as CYP2C8 and CYP2A6 were identified as TMSs by AFTM, but not by UniProt or HTP. Their paralogs have been classified as single-pass TMPs by AFTM, UniProt, HTP and Membranome, suggesting that CYP2C8 and CYP2A6 could also be TMPs. Two mitochondrial ATP synthase subunits ATP5PB and ATP5ME were also identified as TMPs by AFTM, but not by UniProt or HTP. A recent structure of the ovine ATP synthase indeed showed that the predicted TMSs of ATP5PB and ATP5ME (which have 88% identity to their respective human orthologs) are located in the membrane (PDB: 6ZA9) (28). We included 24 such proteins in the AFTM online database and excluded a few that are likely not TMPs (e.g. the transcription factor Myc-associated zinc finger protein with a polyalanine segment predicted as a TMS and the Insulin-like growth factor I (IGF1) protein where the predicted TMS is within a propeptide).

### The online database of AFTM—a comprehensive resource of human TMPs

We built an online database (http://conglab.swmed.edu/AFTM) to display the results of AFTM and compare them to those of UniProt and HTP. The AFTM website features a summary page that presents the numbers of TMSs from the three sources for candidate human TMPs. TMPs supported by TmAlphaFold and PDBTM and single-pass TMPs classified in Membranome are also shown on the summary page. One advantage of the online AFTM database is the integration of the results of various resources (AFTM, UniProt, HTP, TmAlphaFold, PDBTM and Membranome) along with AlphaFold structure models, making it easier to identify human TMPs and their TMSs. Each protein in the AFTM database has its own web page, displaying its primary sequence (in blocks of 100 residues) with lines indicating the location of potential TMSs from various sources (one example shown in Figure 1g). The web page also includes information about the sequence, structure and function of the protein, such as sequence conservation (29), AlphaFold per-residue pLDDT scores (15), AlphaFold-ordered domains, secondary structure predictions by PSI-blast based secondary structure PREDiction (PSIPRED) (30) and SPIDER3 (31), disordered region predictions by SPOT-Disorder (SPOTD) (32) and IUPRED2A (33) and UniProt features such as glycosylation sites, disulfide bonds and functional regions. The results of PPM3 on AlphaFold models (full protein without regions of low pLDDT scores or individual domains) in PDB format with membrane boundaries are available for download. Proteins can be searched by gene names or UniProt accessions, and the entire database of candidate TMPs and their TMSs annotated by different sources is available for download.

## Conclusions

The AFTM database utilizes high-quality AlphaFold structural models to detect TMSs in the human proteome. AFTM results differ from annotations from the UniProt database and the HTP database. In general, AFTM identified more transmembrane regions for multipass TMPs compared to UniProt and HTP but missed some TMSs in single-pass TMPs due to limitations of AlphaFold models. AFTM also suggests that some proteins annotated as TMPs by UniProt or HTP may be non-TMPs. Our results could provide new insights into the structure and topology of human TMPs and have the potential in advancing protein structure-function research and drug development.

## Materials and Methods

### Partition of AlphaFold models into ordered and disordered domains

We parsed proteins into ordered domains and disordered domains based on AlphaFold models (15, 17). In addition to the predicted 3D structure, AlphaFold provides the predicted aligned error (PAE) for each residue pair in a protein, which reflects AlphaFold’s confidence in the distance between two residues, making it useful in defining the domains of a protein. Residues within the same domain are tightly packed together to form a globular and rigid 3D structure, and PAEs of residue pairs inside a domain are expected to be low. In contrast, the relative orientation and distance between different domains might be variable, so a pair of residues from different domains frequently has a high PAE. A disordered segment’s distance from the rest of a protein is also uncertain; thus, it is expected to show high PAEs relative to other residues.

We wrote an in-house script to iterate the following procedure to split any AlphaFold model into segments (domains). A protein or a protein segment is split into two segments if (i) it had >500 residues and (ii) the density of residue pairs showing low PAE (12 Å) within each segment (*D*_intra_) was significantly higher than the density of residue pairs showing low PAE (12 Å) between two segments (*D*_inter_). We found the split site that maximizes the *D*_intra_/*D*_inter_ ratio, and we required this ratio to be at least 10 for proteins or segments >1000 residues and at least 20 for proteins or segments with 500–1000 residues. This process is repeated until all segments are <500 amino acids or cannot be further split.

### Application of PPM3 to identify TMSs in AlphaFold models

The PPM3 program (18) was used to predict the TMSs in AlphaFold models of human proteins. The input of membrane type to the PPM3 program was determined by the subcellular location information in the UniProt entries: mitochondrial inner membrane, mitochondrial outer membrane, ER membrane (mammalian), Golgi membrane, lysosome membrane, endosome membrane, vacuole membrane or plasma membrane (mammalian). For proteins without membrane subcellular location information, the undefined membrane type (empty space) was used for PPM3. For each reviewed human protein in the UniProt database (version: 23 February 2022), we considered it a potential TMP if it has at least one annotated TMS (with the TRANSMEM feature) by UniProt or at least one TMS reported in the HTP database. Regions corresponding to signal peptide and mitochondrial transit peptide according to UniProt were removed from the AlphaFold structural models.

We observed that the random placement of disordered regions relative to globular domains in AlphaFold models often hinders the correct positioning of TMSs in the membrane by PPM3. To address this issue, we thus implemented two approaches. First, positions with low pLDDT scores (<0.5) were removed from the full-length AlphaFold model, and the rest of the model is subject to TMS prediction by PPM3 (AFTM_full). Second, the AlphaFold models were partitioned into domains of ordered regions and disordered regions as described earlier, and each domain was subject to TMS prediction by PPM3 (AFTM_domain). The AFTM method combines the results of AFTM_full and AFTM_domain.

We differentiate the segments reported by PPM3 into transmembrane regions (shown in Supplementary Figure S1a) and re-entrant regions (shown in Supplementary Figure S1b) that enter and exit the membrane from the same side (some of them are annotated as intramembrane regions in UniProt). For each segment reported by PPM3, we extend it to include 10 residues before it and 10 residues after it. The minimum distances among residues in this extended region (using main chain atoms) to both membrane boundaries (defined as two planes or as two spheres in the output of PPM3) were calculated. If both the minimum distances are <10 Å, we define this region to be a TMS. Otherwise, the region is classified as a re-entrant region and is not reported as a TMS by AFTM.

In addition, we merged the consecutive PPM3 segments if they have the same orientation and are separated by <10 residues (shown in Supplementary Figure S1c). This procedure merged some broken or kinked helices into one TMS, which are sometimes defined as two separate TMSs by PPM3.

Finally, PPM3 fails to recognize some TMSs. These cases are indicated by two consecutive PPM3 segments that exhibit the same orientation relative to the membrane and are separated by ≥15 residues (shown in Supplementary Figure S1d). For such cases, we identified the missing TMS in the following way. First, we identified the residue in the region between the two PPM3 segments that is closest to the membrane center (indicated by having the smallest difference between its distances to the two membrane boundaries). Second, this center residue and up to 10 residues around it (provided they do not overlap with the two PPM3-reported TMSs) were considered to constitute the missing TMS, which was added to the set of TMSs defined by AFTM.

### Mapping TMSs from PDBTM and Membranome to human proteins

We mapped the TMS regions reported in PDBTM to the human proteome by DIAMOND BLAST searches (34). Only hits with sequence identity >95% were kept. One human protein may have multiple TMSs from multiple PDB records mapped from the PDBTM database. We clustered these TMSs using single linkage clustering (any two segments with an overlap of ≥10 residues are put in the same cluster). For each cluster of overlapping TMSs, we defined a consensus TMS of PDBTM, with the start and end positions determined by the median of the start and end positions of all these TMSs (round down to integers). In total, 601 human proteins in our dataset have one or more TMSs mapped from the PDBTM database. The same procedure was used to map TMSs in 2322 human proteins from the Membranome database (35), which consists of a dataset of curated single-pass TMPs.

## Supplementary Material

baad008_Supp

## Data Availability

The AFTM database is available at http://conglab.swmed.edu/AFTM.
